# Design and Implementation of an Integrated IoT Blockchain Platform for Sensing Data Integrity

**DOI:** 10.3390/s19102228

**Published:** 2019-05-14

**Authors:** Lei Hang, Do-Hyeun Kim

**Affiliations:** Department of Computer Engineering, Jeju National University, Jeju 63243, Korea; hanglei@jejunu.ac.kr

**Keywords:** Internet of Things, sensing data integrity, smart contract, permissioned blockchain, resource-constrained

## Abstract

With the rapid development of communication technologies, the Internet of Things (IoT) is getting out of its infancy, into full maturity, and tends to be developed in an explosively rapid way, with more and more data transmitted and processed. As a result, the ability to manage devices deployed worldwide has been given more and advanced requirements in practical application performances. Most existing IoT platforms are highly centralized architectures, which suffer from various technical limitations, such as a cyber-attack and single point of failure. A new solution direction is essential to enhance data accessing, while regulating it with government mandates in privacy and security. In this paper, we propose an integrated IoT platform using blockchain technology to guarantee sensing data integrity. The aim of this platform is to afford the device owner a practical application that provides a comprehensive, immutable log and allows easy access to their devices deployed in different domains. It also provides characteristics of general IoT systems, allows for real-time monitoring, and control between the end user and device. The business logic of the application is defined by the smart contract, which contains rules and conditions. The proposed approach is backed by a proof of concept implementation in realistic IoT scenarios, utilizing Raspberry Pi devices and a permissioned network called Hyperledger Fabric. Lastly, a benchmark study using various performance metrics is made to highlight the significance of the proposed work. The analysis results indicate that the designed platform is suitable for the resource-constrained IoT architecture and is scalable to be extended in various IoT scenarios.

## 1. Introduction

The adoption of IoT-based technologies opens up new opportunities in various aspects of our daily lives, such as home automation, intelligent transportation, and manufacturing [1]. With the evolution of embedded computing hardware and network technology, the integration of these two technologies makes large-scale autonomous IoT systems come into being [2]. In general, the IoT system consists of heterogenous devices that produce and exchange vast amounts of safety-critical data, as well as privacy-sensitive information. Therefore, the network is becoming especially critical. Due to working in unattended environments, the wireless sensor network is most vulnerable to a variety of cyber-attacks [3]. Most current IoT solutions depend on the centralized architecture by connecting to cloud servers through the Internet. This solution provides magnificent elastic computation and data management abilities, as IoT systems are growing more complex; however, it still faces a variety of security issues. One of the disadvantages is that the widely-expanded IoT-based infrastructure can introduce a single point of failure, which can compromise the availability of the entire data center. It is necessary to implement a tamper-proof environment as well as a fault-tolerant network for the large number of IoT devices [4].

More suitable solutions need to be proposed, and some researchers started to introduce new paradigms by using a decentralized technology for the IoT device access control, that is blockchain [5]. From a conceptual level, blockchain is a kind of secured, distributed database comprised by numerous peers that are able to track, verify, and execute transactions and store information from a large variety of entities. This idea has already been applied to dream up high-level use cases in various realistic scenarios [6], such as intelligent transport system [7], medical records management [8,9,10], decentralized web applications [11,12], and prediction platforms [13,14]. In short, the main advantages of the blockchain are great transparency, enhanced security, improved traceability, high efficiency, low costing, and no third-party intervention [15]. 

Blockchain technology is a revolution in systems of record and has been foreseen by the industry and research community as emerging technology that can play a significant role in monitoring, controlling, and, most importantly, securing IoT devices [16,17,18,19,20]. The authors describe a blueprint on the combination of IoT and blockchain technologies, which facilitate the sharing of IoT resources and services, and allow the automation of time-sensitive workflows in cryptographically manner [21]. This work identifies solutions and workarounds to highlight that the blockchains and IoT can be used together. For example, data transmitted by IoT devices is always cryptographically proofed by the signature of the sender who holds a unique key pair; therefore, the authentication and integrity of transmitted data are guaranteed. Moreover, all transactions made to or by IoT devices are recorded on the distributed ledger that can be traced back securely. Although the blockchain may look like a panacea to solve IoT security of privacy issues that exist in the current centralized architectures, there are still many research challenges that prevent its incorporation into modern IoT networks. In fact, most consensus algorithms used by current blockchain-based systems are not designed to be run on devices with extreme limitations in computing resources. Proof-of-Work (PoW) [22] is the first consensus algorithm introduced in the blockchain network, and is used by many existing systems. It spreads the responsibility for a decision to all the individual nodes, namely miners. This process is so called mining, which requires massive computing capacities.

The development of embedded devices like smart phones increases much more slowly than desktop systems. As a result, it is difficult for these kind of devices [23] to operate transactions properly using the current blockchain-based systems, in terms of low computing powers and limited data storage. Although there are an increasing number of devices with integrated blockchain capabilities available on the market. For example, embedded devices such as Raspberry Pi and Beaglebone Black are permitted to install the full Ethereum node by EthEmbedded [24]. Furthermore, the use of wallet for Bitcoin and Litecoin are supported by Raspnode [25], thus mining can be done on embedded devices. However, as stated by Raspnode, it would be useless to perform mining on IoT devices. Alternatively, some hardware such as application-specific integrated circuit (ASIC) chips are designed and manufactured for mining, since it is improper to try it on IoT devices [26]. There is still a lot of research to be done for enabling a wide integration of IoT devices as blockchain components.

Therefore, new ways of solving these issues are needed when considering the integration of blockchain with IoT, for instance, one of the primary challenges is how to adapt the blockchain technology that suited to embedded IoT devices with limited resources. A new or a customized implementation of blockchain is required as different applications have different requirements. By contrast to other existing system proposals, the proposed approach in this paper brings the advancements in IoT as follows:

1. Scalability: Our solution meets the requirements of the practical IoT network, which is comprised of numerous IoT devices connected, through different constrained networks, to a single blockchain.
2. High throughput: A high throughput network is needed so as to deal with simultaneous communications among a large variety of devices. This work proposes the use of a permissioned blockchain, in which interactions occur among a set of network entities that fully trust each other. As a consequence, traditional voting-based protocols, like byzantine fault tolerant (BFT) or crash fault tolerant (CFT) consensus protocols, can be used to improve the network throughput.
3. Lightweight: In our solution, the IoT devices are not included in the blockchain, and alternatively, a RESTful interface which handle requests from devices is defined to enable cross platform communication between devices and the blockchain network.
4. Transparency: This system hides the details of the IoT devices and the transaction history that records how a resource is manipulated, except for to the authorized user.

More precisely, we present a decentralized scheme in which device information could be stored on a secure, permissioned chain and shared back and forth quickly, like email. The proposed architecture is extended from the IoT platform in our previous work [27]. In order to ease the interaction between the end user and the blockchain network, various interfaces are implemented by utilizing some of the web front-end technologies, such as JavaScript and HTML5. This web-driven paradigm allows end users to improve the access and management of the resources within the blockchain network. All the product-specific services provided by the blockchain network are exposed as representational state transfer application programming interfaces (REST APIs), which can be invoked by either web clients or IoT devices. Device users can control and be aware of the surrounding environment without a priori knowledge of the physical devices, for example, the types of physical devices and how to set up them. These IoT devices do not need to install the full node, since the consensus process, such as the practical byzantine fault tolerance (PBFT) algorithm, is performed in the blockchain network from a remote space. Smart contract is used to provide controlled access to the device meta-data and to host the ledger functions across the network. In the designed platform, we also define access control policy, which allows participants to access a certain number of contents or transactions that are authorized. For example, only the owner of the device is permitted to access and manipulate the device. Since blockchain technology is not intended for large transaction data payloads so that new data storage technologies are desired to deal with a large amount of IoT data. In our system, we apply a separate software solution by deploying the Couch database (DB), resided on each peer, to enable the large file storage and minimize the duplication across the entire blockchain filesystem. Lastly, we prove the practicability of our proposed approach by implementing a real-life case study in a smart space. The blockchain network is implemented by using the Hyperledger Fabric [28], which is a permissioned decentralized platform designed for building decentralized applications (DApps) or distributed ledger solutions on top of it. 

The remainder of this paper is structured as follows: Section 2 provides an overview of a number of the related projects and analyzes some common issues in current state of art. Section 3 looks into the system architecture and demonstrates the work flow of the proposed IoT blockchain platform. Section 4 explains in-depth about the implementation of the smart space case study and presents execution results with various snapshots. Section 5 presents the evaluation results of the proposed platform in various performance metrics. Section 6 highlights the significance of the proposed work through a benchmark analysis, by comparing the designed approach with some recent studies. Section 7 concludes the paper and discusses future research directions.

## 2. Related Work

So far, IoT technology has been widely adopted by the manufacturing industry in machine-to-machine (M2M) communication. While the current technologies make the concept of IoT feasible, a large number of challenges lie ahead for aiding large-scale real-world deployment of IoT applications. In recent years, the blockchain has attracted extensive attention from researchers and companies for its security and transparency. The blockchain has great potential to be the organizational structure for interconnecting everything and for timestamping heterogeneous data in Industry 4.0 [29]. The common theme in this paper is the collaboration of blockchain and control of IoT. To the best of our knowledge, research works on this theme is limited because blockchain is quite well-known in financial services. In this section, we explore those blockchain technologies involved in IoT by overviewing some recent studies. 

A total of 18 cases of blockchain use have been categorized [30], and four of them are specified to IoT, including immutable log of events and access management of data [31], sensing data trading [32], IoT equipment trading [33], and IoT devices authentication [34]. The authors discuss the integration of the blockchain with IoT and highlight the integration benefits, challenges, and future directions [35]. Mainly due to its decentralized features in computation and management processes, the blockchain can be a powerful technology to solve many IoT issues, especially security. They elaborate that it is the right way to move the current centralized IoT system towards the decentralized architecture. A built-in blockchain solution is proposed [36] for LoRaWAN network servers in order to implement an open, trusted, decentralized, and tamper-proof system. An incontrovertible mechanism is provided to verify the data of a transaction that existed at a specific time in the network. The authors declare that this work takes advantages of the blockchain technology and it is the first implementation to integrate blockchain with LoRaWAN IoT technology. Another proof of concept using LoRa nodes is proposed [37] to enable low-power, resource-constrained IoT end-devices to access a blockchain-based infrastructure in an Ethereum network. To achieve this aim, the authors utilize an IoT gateway as a blockchain node and propose an event-based messaging mechanism for low-power IoT end devices. The authors review the usage of smart contracts in IoT and describe how smart contracts can facilitate and support the autonomous sharing of services among IoT devices [38], as proposed in [39]. The significance that IoT can profit from blockchain networks are described in terms of trading, billing, shipment, and supply chain management. The authors present a traceability system for tracking Chinese agri-food supplies [40]. The proposed system combines the radio frequency identification (RFID) with blockchain technology to enhance food quality and safety but meanwhile to reduce transportation costs. An IoT device management system is proposed [41] to control and configure IoT devices remotely. The authors propose a refreshing key management scheme whereby public keys are saved in Ethereum while private keys are stored on each IoT device. The Ethereum network is used in the proof of concept since it provides the means to model smart contract than can be run on top of the network. In this way, the maintenance and debugging are simplified since the update of code can directly change the behavior of IoT devices. A smart city application framework is proposed to integrate heterogenous smart devices in a highly secure manner [42]. The proposed framework provides a variety of features, including better fault tolerance capability, improved reliability, scalability, and efficient operation, which set up a common blockchain eco system in which all devices could communicate with each other in a secure distributed environment. The authors propose a lightweight architecture for IoT to eliminate the overheads of classic blockchains, while maintaining most of its security and privacy benefits [43]. A private immutable ledger that is managed centrally is designed to optimize energy consumption from IoT devices. In addition, they use the distributed trust to reduce the block validation processing time. Lastly, a representative case study specified for smart home is implemented to explore the usability of the proposed architecture. CONNECT [44] is a theoretical blockchain-based architecture that concentrates both on IoT service provision and heterogenous device interconnection. The proposed architecture takes advantage of hierarchical and multi-layered blockchains, which enable the building of a contextual service discovery mechanism. FairAccess [45] is a fully decentralized management framework based on blockchain that enables users to own and control device data. In this framework, new types of transactions are designed to issue and revoke the access using smart contracts. A decentralized, cloud-based platform specified for industry manufacturing on the basis of blockchain technology is proposed [46]. The authors build a trusted intermediary for transactions among the users in order to provide on-demand access to manufacturing resources. Single board computers, such as Beaglebone Black and Raspberry, are utilized for communication between machines, the cloud, and the blockchain network. The authors propose a decentralized system, allowing sensors to exchange Bitcoins with data [47]. To be more precise, the client requests data by sending the transaction (with Bitcoins) to the address of the specific sensor, in turn the sensor responds to the client with sensing data. To ensure the device ownership, the authors design a blockchain-based layer storage system in order to give the end users full control over their devices [48]. The proposed system consists of three layers: data layer, control layer, and device layer. The data layer collects IoT data and provides the decentralized hash table for data storage. The control layer manages the access control on the data stored in the hash table. The device layer exposes various services provided by IoT sensors and actuators. Sapphire [49] is a novel system that is aiming to exploit the computing power of IoT devices to perform computation on collected data by using smart contracts. The results indicate that this approach can reduce the data transfer on the IoT network and improve the transaction execution. The authors present a decentralized system to preserve user’s privacy on IoT devices (Bluetooth low energy modules) by using the Ethereum platform [50]. Besides, a blockchain gateway is introduced to interact with the IoT towards the user. Two types of smart contracts are proposed: one for the IoT device and the other for the gateway. The authors deploy the blockchain technology into cloud architecture to enhance data transparency and decentralization [51]. The proposed platform abstracts the physical resources into the cyber space and exposes them as services. Essentially, blockchain is used as a middleware between the cloud and the manufacturing resources, guaranteeing the transparency and security of the data in cloud manufacturing.

As mentioned, these studies have some common issues that are inappropriate for resource-constrained IoT devices, such as token-based transactions, which cause the high time cost involved in transaction executions. The nonuse of native cryptocurrency can remove the risk of virtual currency speculation, and can roughly obtain the same processing performance as any other distributed system. Furthermore, they deploy the copy of ledger onto embedded devices that act as nodes of the blockchain network. In such way, the transaction execution rate is heavily impacted and it is hard to fulfill the requirement of a global IoT ecosystem with millions of nodes. For the need of practical application, this paper gives feasible solutions that are extensible to large-scale networks with low latency and high throughput. In addition, the implementations that are compatible with resource-constrained IoT devices are also presented.

## 3. Designed Architecture of the Proposed IoT Blockchain Platform

### 3.1. Conceptual Scenario of the IoT Blockchain Platform

**Figure 1:**
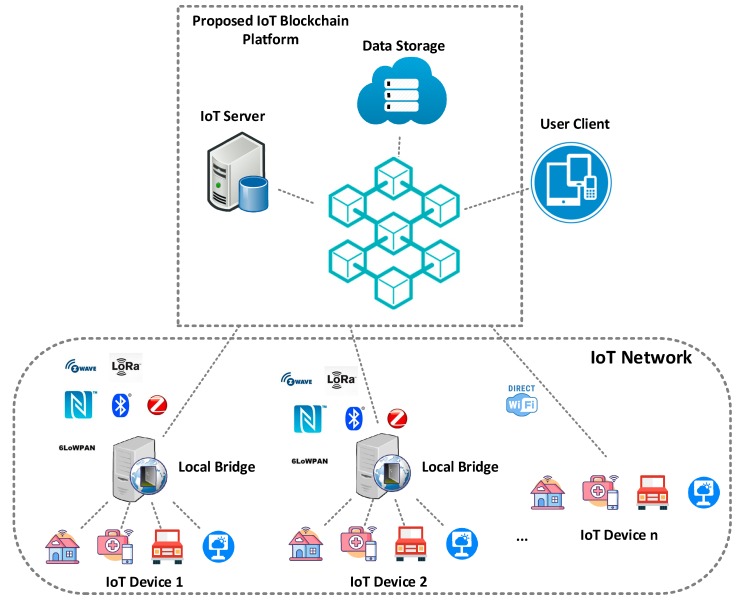
IoT blockchain platform conceptual scenario.

Figure 1 represents the conceptual scenario of the IoT blockchain platform, which comprises of a massive number of IoT devices, data storages, user devices, servers, and local bridges that are linked together around a peer-to-peer blockchain network. The IoT server is a service provider that can interact with the local bridges and the blockchain network to provide a large variety of services for end users, such as collecting sensing data from the bridge, sending commands to perform some operations on the actuators, querying data, or storing data to the storage space via the blockchain network, etc. The data storage that resides in the blockchain network can store physical device profiles, environmental data collected by sensors, and device owner profiles. It can either be a hardware storage like a hard disk or a software storage such as a DB. User client can be any terminal devices, such as smart phones, laptops, and PCs, through which end users can read or write data to the blockchain network. For example, home users can view the status of different home appliances that are stored in the blockchain at a specific period. There exists a wide choice of communication protocols for developers to apply on products and systems in IoT, such as Bluetooth, ZigBee, WiFi, and 2G/3G/4G cellular. Local bridges connect a cluster of IoT devices to the server through these communication technologies and act as the service agent for these devices as well. Nowadays, with the advancement of hardware technology, embedded devices such as Raspberry Pi can directly consume web services by invoking representational state transfer application programming interfaces (REST APIs). Therefore, two approaches are presented for communicating with physical devices, that is either via the local bridges or via direct wireless communications. Unlike most existing projects that focus on the use of bridge to connect IoT devices with the blockchain network, the proposed work concentrates on the communication in a straightforward manner. The IoT devices can be classified into sensors and actuators: sensors are used to collect environmental data such as temperature and send these data to servers for further usage, while actuators are used to perform particular actions (e.g., turn on the light) according to commands received from end users.

### 3.2. Proposed IoT Blockchain Platform System Architecture

**Figure 2:**
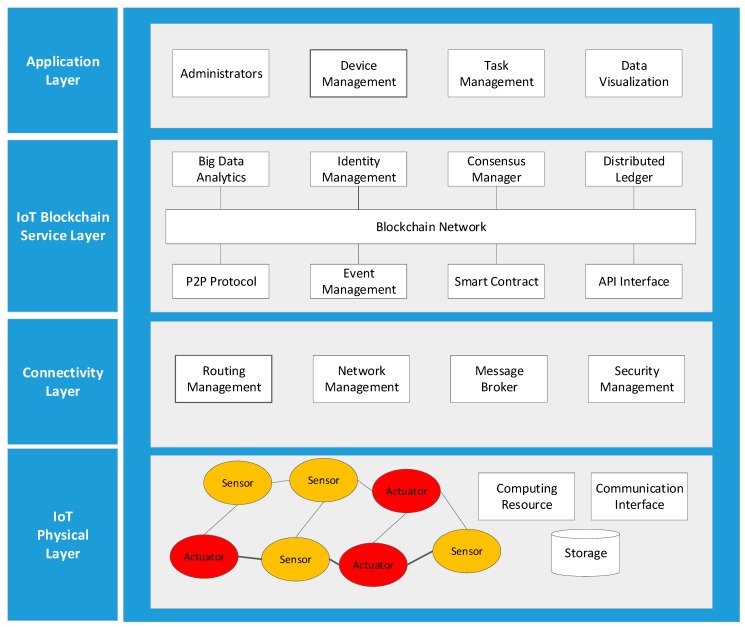
Layer-based IoT blockchain platform architecture.

Figure 2 presents the layer-based architecture of the proposed IoT blockchain platform that is extended from our previous work [27]. It is a modular architecture whereby each layer is decoupled from other layers so that developers can replace or add any new module without affecting the rest of the system. The IoT physical layer consists of various linked devices with the abilities of communication, computing, and data storage. The main function provided by the connectivity layer is routing management, because self-organization is required since physical devices themselves have no global internet protocols (IPs). This layer also contains other modules for providing services, including network management, security management, and message broker. The IoT blockchain service layer contains all modules that organize common services to provide various features of blockchain technologies, including identity management, consensus, and peer-to-peer (P2P) communication. The distributed ledger is a consensus of replicated, shared, and synchronized digital data that spread across the whole blockchain network, where all participants with the network can have their own selfsame copy of the ledger. It also provides secure storage space to record the device configuration and sensing data provided by physical sensors. Any changes to the ledger are reflected in all copies in minutes, or in some cases, seconds. The ledger can be either permissioned or permissionless, regarding if anyone or only approved members can run a peer to validate transactions. The big data analytics module enables the blockchain to be an efficient mode for online data storage. Lots of transactional data from various parties are stored in structured forms of ledgers, which makes it a perfect source for further analysis. All of these parties can be granted access to one single network and it will be convenient to access these details. The smart contract is sort of code invoked by an external client application to manage access and modifications in the ledger. It is usually installed and instantiated onto every peer of the network. The event management sends events every time a new block is added to ledger or triggered whenever the predefined condition in the smart contract is fulfilled. The API interface exposes the services provided by the blockchain network as services through which the client application can access and manage the network. The top layer is the application layer, where various interfaces are provided to visualize the data from physical devices, to manipulate and control devices.

### 3.3. Interaction Model for the Proposed IoT Blockchain Platform

**Figure 3:**
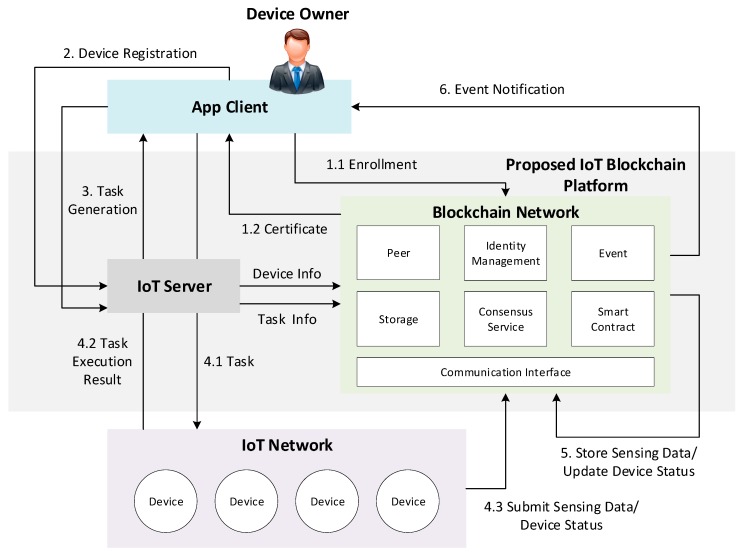
System work flow of the proposed IoT blockchain platform.

Figure 3 describes the system work flow and gives a solid understanding for each component of the proposed IoT blockchain platform. The designed platform encompasses not just a technical infrastructure but also a user service framework that exposes the distributed ledger and smart contract as services to applications. The app client provides an intuitive interface to submit transaction proposals to the blockchain network for consuming services such as user enrollment, device registration, and task generation services provided by the blockchain network. Before submitting a transaction, the enrollment is required to supply a specific participant with certificate, which contains private keys to sign the transaction. A transaction can be defined as a process of reading or writing data from the ledger that is performed among the blockchain network. The device owner can submit transaction to register a new device or generate a new task through the IoT server. In turn, the server transfers the request to the blockchain network to perform some certain operations. It can also transfer the task request from client to the device and send back collected sensing data/ status changes from the device in real time. Since the identity of the device owner is authenticated, the physical device associated with the specific owner can directly submit transactions (including owner info) to the blockchain network. The sensing data or status is then appended in the ledger and compared with the threshold defined by the smart contract. If the value exceeds the threshold level, a notification will be generated to alert the device owner.

**Figure 4:**
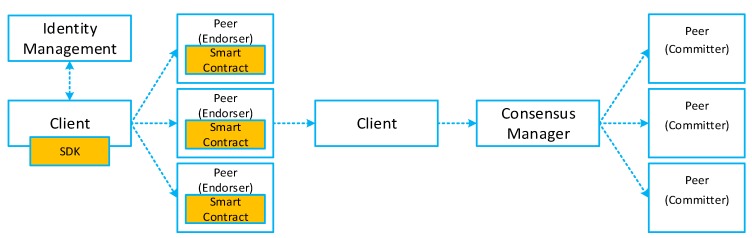
Detailed transaction execution work flow.

Figure 4 illustrates the detailed transaction execution process taken place in the blockchain network. The client application must have credentials issued by the identity management service so as to get the authorized permission for submitting transaction proposals. The identity manager holds user IDs and authenticates clients who want to enroll in the network. Transactions start out from client applications sending transaction proposals to peers in the blockchain network. The communication between the blockchain network and the client application happens over the application software development kit (SDK). These peers can either be endorsers or committers: Endorsers cam simulate and sign transaction proposals, respond to granting, or deny approvals; while committers validate transaction results prior to the writing of a block of transactions to the ledger. Each endorser peer receives and executes the transaction proposal by invoking the smart contract in their own simulated environment. It is worth noting that in this stage, the execution results will not be reflected in the ledger. These endorser peers just capture the set of Read and Written data, namely RW sets, which record what was read from the current state when the transaction is simulated and what would write to the state after the transaction has been executed. Each endorser peer signs the RW sets and returns proposal responses to the client application for endorsement inspection. The client verifies the endorsing signatures to determine if the specified endorsement policy (the set of peers that must endorse the smart contract execution results) has been fulfilled. Then the client packages the signed transaction and submits this transaction along with RW sets to the consensus manager. Consensus happens across the network, in parallel with signed transactions, and RW sets are submitted; this information is ordered into a block and delivered to all committer peers. Each committer peer validates the transaction by checking whether the RW sets match the current state. Specifically, the Read data still exists even if the transaction simulated by the endorser is identical to the current state. After the committer peer validates the transaction, the transaction is written to the ledger, and the state is updated with the Write data from the RW set accordingly. Lastly, the committer peers asynchronously notify the client application as to whether the submitted transaction succeeded or not. Client applications can register for events so that they can be notified by each committer peer when events occur.

### 3.4. Smart Contract in the Proposed IoT Blockchain Platform

The concept of the smart contract [52] was first introduced by Nick Szabo in 1994, which is defined as “a computerized transaction protocol that executes the terms of a contract”. Within the context of blockchain, the smart contract acts as a trusted distributed application that gains its trust from the blockchain and the underlying consensus among the peers. Since they reside on the blockchain, smart contracts have a unique address through which the end user can address a transaction to it. According to the data that triggers the predefined condition, the smart contract then executes automatically and independently in a prescribed manner by every peer in the network.

**Figure 5:**
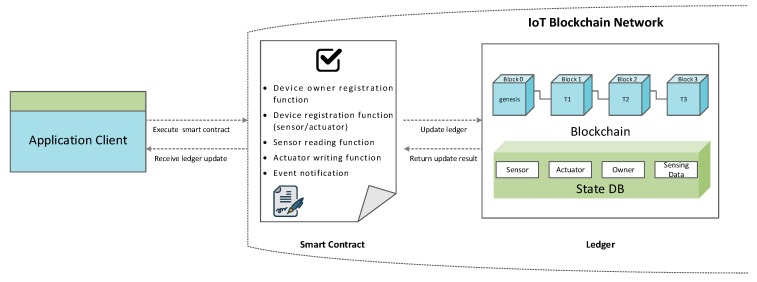
Smart contract for IoT blockchain network interaction.

In principle, smart contracts are written in a non-standard, or domain-specific language (such as Solidity) in order to reach consensus among all of the peers. This becomes one of the greatest challenges to the wide-scale usage of smart contract, since blockchain developers must learn a new language to write smart contracts, and this may lead to various problems in coding. Moreover, transaction execution performance and scale are limited since all transactions are executed successively by all peers. To address these issues, we deploy smart contracts onto a specific subset of peers rather than to all peers, hence, the transaction only needs to be executed by a set of peers. This approach also supports parallel execution, which can prominently increase the overall performance and scale of the system. Furthermore, we use the standard languages such as Node.js or Java to code the smart contract so that developers can use their familiar programming languages without spending time learning a new language. As shown in Figure 5, the proposed smart contract contains various functions that allow users to interact with the ledger, which is a combination of the state database and blockchain. For example, users can create, update, and query device information from the ledger by submitting transactions to the smart contract. It also provides functionalities to handle the transactions proposed from the devices, such as collecting sensing data or updating state changes of actuators. A block contains a hash value of the transactions and the hash value of the prior block in order to insure the security of the ledger data. Even though the ledger hosted by one peer is tampered with, it would not be able to convince all the other peers because the ledger is distributed throughout a network. A sample structure of a ledger is given in Figure 5, where the blockchain contains four blocks. Block 0 is the genesis block, which does not contain any transactions. Each of the other blocks contain one transaction, and these transactions are associated with various assets (e.g., sensor, actuator) in the ledger state. The application running on the smart contract receives the transaction and performs different kinds of queries and updates. The transaction is appended in the block, and meanwhile the ledger state is updated. In the end, the ledger updating result is returned to the application as the response.

### 3.5. Proposed IoT Blockchain Platform Execution Procedure

**Figure 6:**
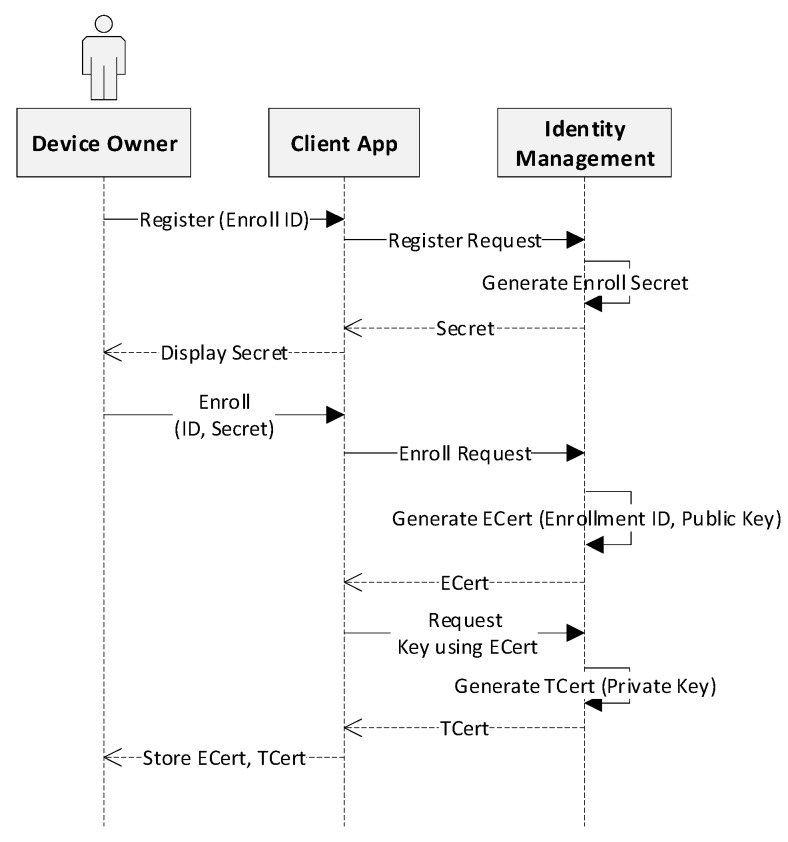
Identity issue for network user.

As mentioned above, the network user must have credentials before they are permitted to submit transaction proposals to the blockchain network. Therefore, the system execution procedures are classified into two sequence diagrams, respectively. Figure 6 represents the processes of identity registration and enrollment for the device owner. In order to obtain the identity, the device owner submits the registration request to the blockchain network. This request is handled by the identity management module, which issues a secret for the enrollment process through the client app. Enrollment request is then sent from the client, passing the enroll ID and secret obtained in the registration process. The identity management service passes the enrollment certificate (ECert) along with the public key for response. The ECert is used to request for the transaction certificate (TCert), and finally the TCert is returned for signing the transactions.

**Figure 7:**
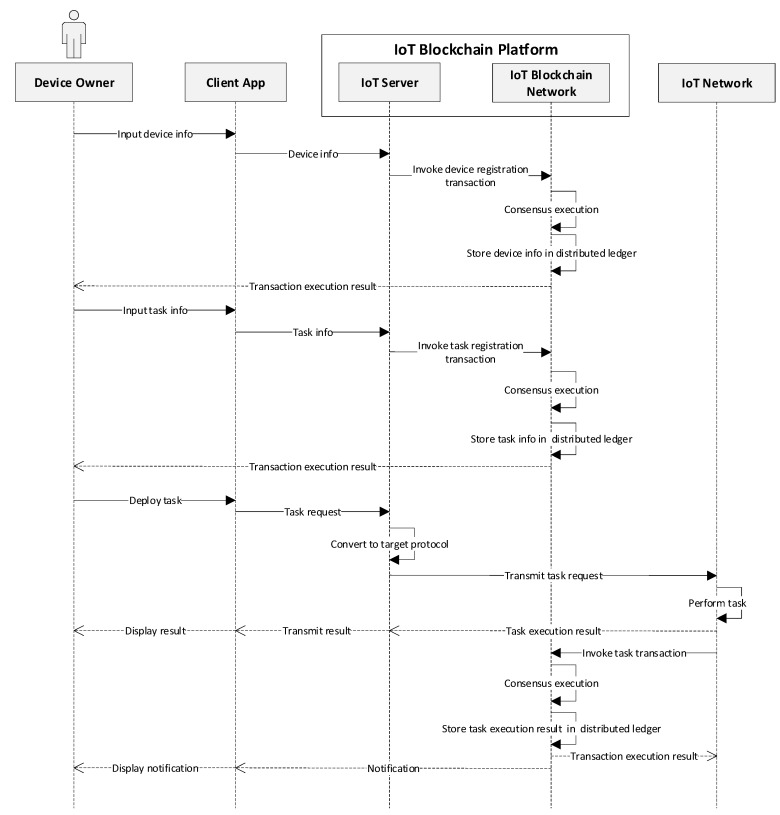
Sequence diagram of various operations within the proposed system.

After the enrollment, the device owner is allowed to access and consume services provided by the network. Various operations happen among different components within the designed platform, which are presented in Figure 7. The device owner inputs information of the IoT device through the client application to register a new device. This information is sent along with the request and the server, in turn, invokes the device registration transaction defined by the smart contract. The consensus process is then executed in the blockchain network, where each peer appends the transaction into blockchain and stores the device information in the state database. After updating the ledger, a response is initialized to inform the client that the transaction is executed. Similarly, the device owner can generate tasks that are used for performing some operations (e.g., read temperature from the thermometer) on particular devices. The device owner can deploy a certain task to a specific device through the client. The IoT server converts the request into the specified protocol of the device and transfers the task info to that target device. The target device in the IoT network performs the task accordingly and returns the execution result (e.g., temperature value) to the server as well as to the blockchain. The execution result forwarded to the server is displayed to the device owner in the client immediately. For the other one, the execution result is packaged as a payload of the transaction and sent to the blockchain network. The blockchain network appends the task execution result to the distributed ledger of each peer and responds the execution results to the device. It also generates the notification to alert the device owner whenever the predefined condition is triggered, for example, the current temperature exceeds the threshold defined by the owner. 

## 4. Implementation of the Proposed IoT Blockchain Platform

### 4.1. Development Environment

The proposed platform consists of three parts as shown in Figure 3, so that the development environments are summarized into three tables to describe each part, respectively. The technology stacks for implementing the IoT blockchain network in docker environments are depicted as shown in Table 1. The operating system is Ubuntu Linux 18.04 LTS with Intel Core i5-8500 @ 3.00GHz processor and 12 GB memory. Docker engine (version 18.06.1-ce) provides the docker running environment and docker-compose (version 1.13.0) provides the integrated development environment (IDE) to configure docker images and containers in the virtual machine. We used the Hyperledger Fabric (v1.2) project, which is an open-source blockchain framework hosted by the Linux Foundation. The Fabric network utilizes Node to develop the client software development kit (SDK) so that Node (v8.11.4) is installed. The web playground provides an interface to design and implement the smart contract definition that contains existing assets and related transactions. Couch DB is used to hold the current values of a set of ledger states. The Composer command line interface (CLI) tool enables developers and administrators to deploy and manage smart contract definitions. REST APIs are generated by the REST server, exposing the blockchain logic to web or mobile applications. 

Table 2 describes the development tools and technologies for implementing the IoT device server that resides on the Raspberry Pi. Android Things is installed on the Raspberry Pi so that the application can be easily programmed in Java language, like a regular Android application. The communication between the device server and the IoT server uses the constrained application protocol (CoAP), while for the communication between the device server and the blockchain network, HTTP is used. Physical resources, such as temperature sensor, humidity sensor, and two LEDs, are abstracted into CoAP resources as part of the server. Each resource is assigned with a unique URI in order to be identified by the server.

Table 3 presents the development stacks to implement the blockchain web application. The application that hosted on the Apache Tomcat can be divided into backend and frontend, which are implemented by Eclipse Photon and WebStorm, respectively. For the backend, the Californium CoAP framework is used to implement the server that translates the communication protocol between web application and IoT device from HTTP to CoAP, and vice versa. For the frontend, a variety of web techniques such as HTML, Cascading Style Sheets (CSS), and JavaScript are used. We make use of Bootstrap and jQuery, which are two popular open-source frontend toolkits for web frontend development. Notify.js is another jQuery plugin to provide customizable notifications to the client. The client can interact with the REST server through which the end user can invoke relevant APIs to submit transactions by HTTP requests using GET or POST.

### 4.2. Use Case Implementation and Deployment

**Figure 8:**
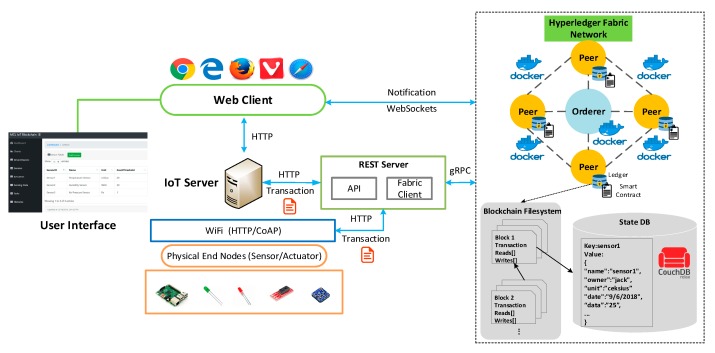
IoT blockchain platform implementation and use case deployment.

Figure 8 illustrates the implementation environment for the case study, and also presents the means of connection between the IoT devices, the server, and the blockchain network. The IoT device server is hosted on the Raspberry Pi, which is integrated with various physical sensors and actuators, that are apparent in the figure. We utilize the Hyperledger Fabric framework to construct the blockchain network, where four peers and an orderer node are running as images in the docker container. Each peer contains the smart contract and data storage to write a block of transactions to the ledger. Couch DB is used as the state database that provides rich query support and the smart contract data is modeled as JavaScript Object Notation (JSON). It supports various query methods such as get, put, and delete in conjunction with a state key, which enables the application to invoke a smart contract to access world states through simple APIs. The example in Figure 8 shows ledger states for one sensor, containing a key and a value. The Couch DB supports a simple state value with only one key-value pair, and a complex state value with multiple key-value pairs as well. In contrast to the state database, the blockchain is physically implemented in a file, as the blockchain data structure is always used to record a limited small set of simple operations. The REST server provides various RESTful APIs that expose the functions defined by the blockchain network. All these services can be invoked by either a web client or a physical device directly. It also hosts the Fabric client, which utilizes Google remote procedures calls (gRPC) system to communicate with the Hyperledger Fabric network. The blockchain acts as a transaction log that records all the state changes. Transactions are; therefore, collected into blocks that are cryptographically linked together to form a sequence of chains, where all transactions on the ledger are sorted in time order, enabling the user to know the history changes that happened in the state database. The orderer node is employed with the PBFT algorithm to ensure the consistency of every copy of the leger. This node exists independently of the peer processes and orders transactions on a first-come-first-serve basis across the network. The notification generated from the blockchain network is emitted to the client using WebSockets.

### 4.3. Smart Contract Modeling for the Case Study

The smart contract is designed and implemented by using the Hyperledger Composer [53], which is an extensive, open development toolset and framework to facilitate the implementation of blockchain applications. Participants are members of a business network, with the ability to have assets and submit transactions. In the proposed case study, they can be device owners who have the ability to access and manage their devices. In general, assets can be goods, services, or property, and are stored in registries. These two types are modeled with an identifier and can have any other properties as required. For example, as represented in Table 4, a device asset that represents the IoT device is summarized into sensor and actuator, typically containing general information such as ID, name, and owner of the specific physical device.

As part of the smart contract, we define the transactions that can interact with assets. Participants can interact with them and each of which can be associated with an identity, across multiple blockchain networks. The condition defines which users are permitted to perform create, read, update, or delete operations in the blockchain network. As given in Table 5, for example, only the device owner of the device can perform update operations on the instance of the device asset. Events are defined in the same way as assets or participants. Once events have been defined, they can be included in the transaction processor functions to be emitted as part of a transaction. Table 6 presents a sample of events defined in the smart contract.

**Figure 9:**
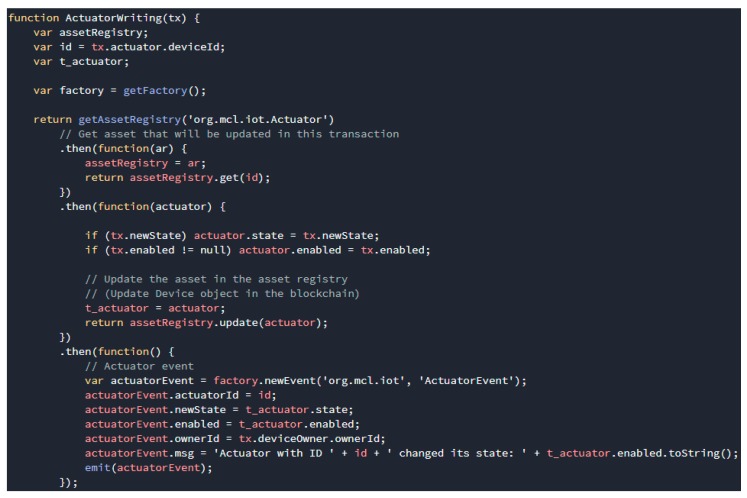
Transaction processor function for actuator writing transaction.

The transaction process function is the logical operation of a transaction defined in the smart contract. As shown in Figure 9, the structure of the transaction processor functions contains a JavaScript function. Here is an example, the transaction processor function relating to the actuator writing transaction is to update the status of the actuator asset. More precisely, this function replaces the status of the actuator asset with the value passed from the physical device, updates the actuator asset in the registry, and then emits an event.

**Figure 10:**
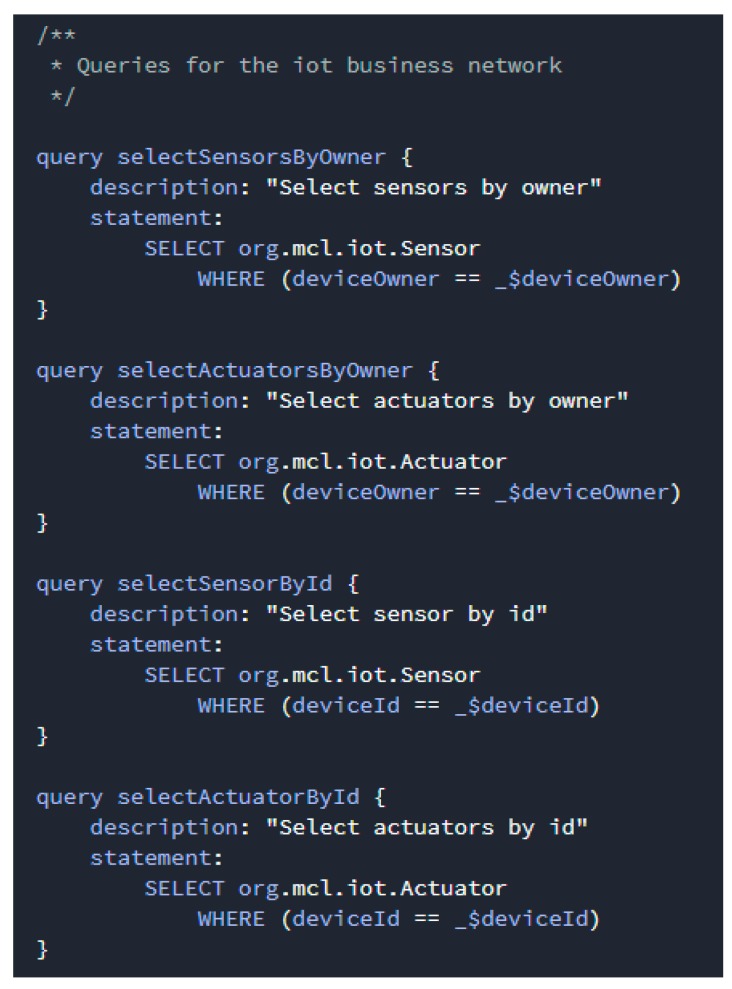
Query definition in smart contract.

Queries are written in a bespoke query language and are defined in a single query file within a smart contract definition. By using queries, data can be easily extracted from the blockchain network. As shown in Figure 10, queries contain a description and a statement, where the query descriptions are a string that describe the function of the query and the query statements contain the operators and functions that control the query behavior. 

Table 7 summarizes a part of REST APIs generated by the composer-rest-server for communication between the web client, IoT device, and the blockchain network. HTTP-based RESTful APIs contain a base URI, a media type that defines state transition data elements (e.g., Application/json), and verbs (e.g., GET, POST, PUT, DELETE). The URI typically represents the path of the data entity, and the verb indicates the desired action to be performed in the identified resource along with the request. For example, a GET request to a resource URI such as /api/Sensor would return a list of sensor information, while a POST request to the same URI would ask the server to accept the entity enclosed in the request.

### 4.4. Execution Process and Results of the Smart Space Case Study

**Figure 11:**
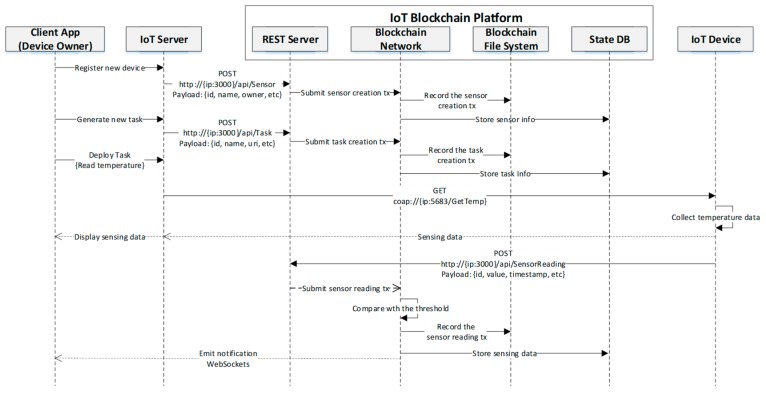
Execution process for the implemented case study.

The execution sequence of the case study is described in Figure 11. First off, the device owner inputs the information of a new device through the client and in turn the IoT server requests the REST server using the POST method. The device information contained in the request is ingested by the blockchain network. The device information is stored in the state DB and the related transaction is recorded in the blockchain file system. Similarly, the task can be generated by the device owner in the blockchain network. The device owner can issue a task request (e.g., read temperature) to the target device and this request is first fetched by the IoT server. The server parses it in the respective format and then passes it to the designated address of the device (e.g., temperature sensor). The device collects the temperature data from the sensor and responds the sensing data to the server. Then the server visualizes the data to the device owner in the client. At the same time, the device submits a sensor reading transaction to the blockchain network by invoking the related API. The blockchain network records the transaction in the blockchain file system and stores the sensing data in the state DB. It also emits the notification to the client via WebSockets, since the sensing value exceeds the threshold defined by the smart contract. 

**Figure 12:**
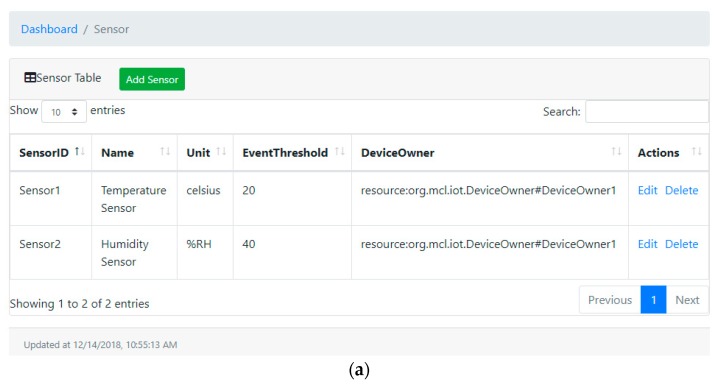

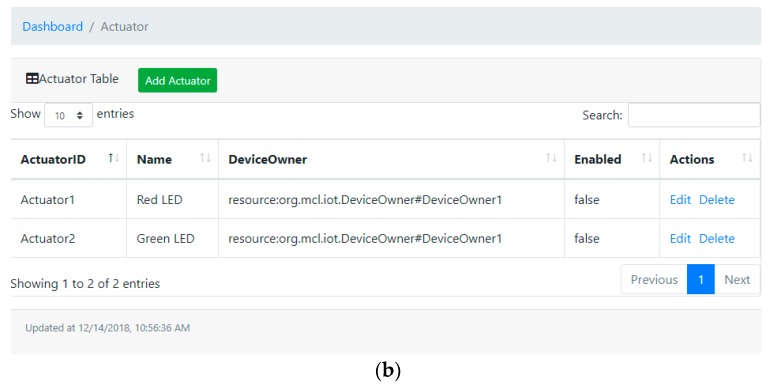
Snapshot of device dashboard. (**a**) Sensor dashboard; and (**b**) actuator dashboard.

Various snapshots of user client are overviewed with the RESTful APIs represented in Table 7 in conjunction with their responses. The client can initialize a request to the REST server for submitting the transaction to the blockchain network. The network invokes the corresponding functions in the smart contract to perform the transaction and returns the response to the client when the transaction is executed. Figure 12 represents the web dashboard used to register and manipulate IoT devices. The IoT device can be either sensor or actuator; therefore, two dashboards are implemented, respectively. 

**Figure 13:**
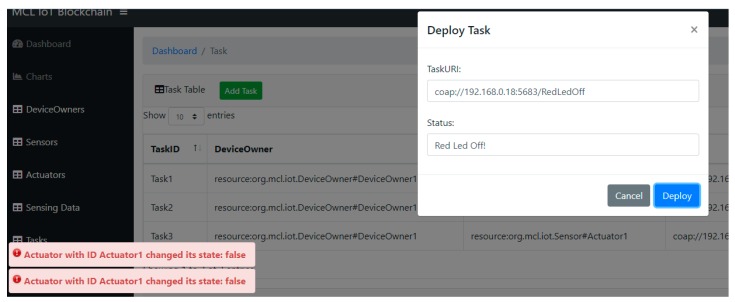
Snapshot of task dashboard.

The snapshot of the task dashboard, where the device owner can generate and allocate IoT tasks is presented in Figure 13. Each task contains a URI that stands for the endpoint of the service exposed by the IoT device. The device owner can deploy the task to the specific device, and after confirming the operation, the request is sent to the physical device. For example, to turn off a red LED, and in turn, the dashboard displays the notification generated from the blockchain network to inform that the device status is changed accordingly.

**Figure 14:**
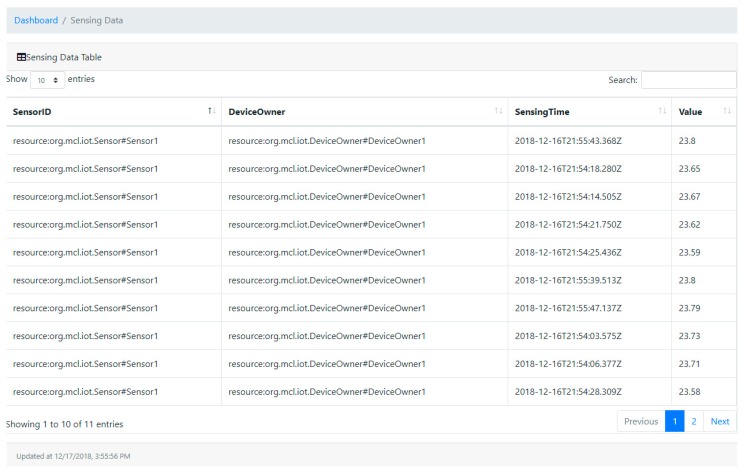
Snapshot of the sensing log dashboard.

Figure 14 represents the snapshot of sensing log history in a timed sequence. Sensing time is an unalterable blockchain ledger record time indicating when the sensor reading transaction is submitted. The value presents the numerical values of readings from the physical device, in this case, this value represents the temperature value.

## 5. Performance Evaluation and Analysis

**Figure 15:**
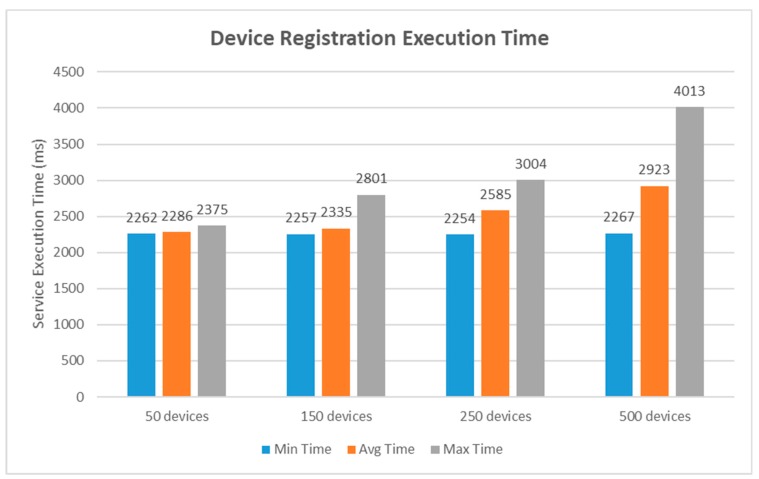
Performance analysis graph of device creation.

This section offers actual evaluation results to assess the performance of the proposed IoT blockchain platform. Several experimental tests were carried out using different performance metrics in order to provide a comprehensive manner. The service execution time included the time for a transaction request to be sent plus the length of time it takes for an acknowledgement to be received by the web client. For this test, we utilized the Postman, which is a tool to dissect RESTful APIs. It provides a sleek user interface to customize scripts for simulating a heavy load on network. The first study was analyzed for the service execution time cost on device registration and the results are shown in Figure 15. For this study, four groups of 50, 150, 250, and 500 devices information were provided to the proposed platform. This was implemented by using the simulation tool called Hyperledger Caliper [54], which allows users to configure the use case script of a specific blockchain implementation with a set of indicators. The execution time taken by the proposed blockchain platform to perform this transaction were recorded in minimum, average, and maximum time. For the 50-device group set, the minimum time was recorded to be 2262 ms, averaging at 2286 ms, and the maximum time was recorded to be 2375 ms. For the 150-device group set, the minimum time taken was recorded to be 2257 ms, averaging at 2335 ms, and the maximum time was recorded to be 2801 ms. For the 250-device group set, the minimum time was recorded to be 2254 ms, averaging at 2585 ms, and the maximum time was recorded to be 3004 ms. Lastly, for the 500-device group set, the minimum time was recorded to be 2267 ms, averaging at 2923 ms, and the maximum time was recorded to be 4013 ms. 

**Figure 16:**
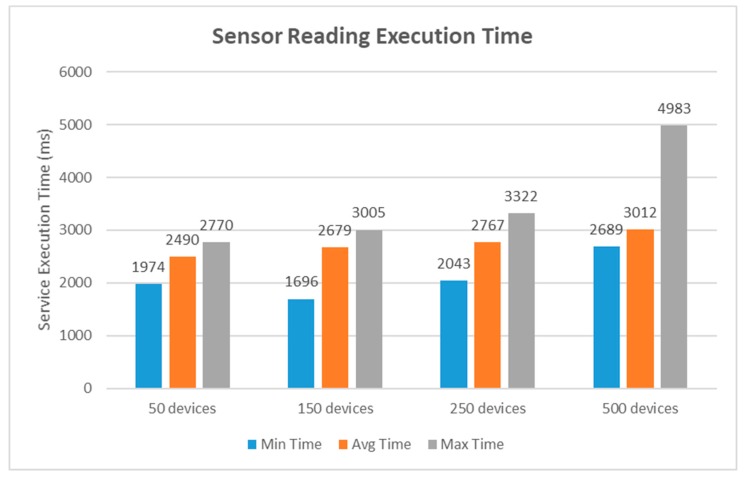
Performance analysis graph of the sensor reading.

In our second study, we evaluated the service execution time on storing sensing data in the blockchain network. All the devices had a HTTP client that was able to request the sensor reading API from the REST server. Once the sensing data was appended in the blockchain, the REST server fetched the execution results from the blockchain network and returned the response to the device. The evaluation results of execution time on performing sensor reading transaction are reported in Figure 16. In both scenarios, the experimental tests were performed by a number of concurrent clients, and every test was measured ten times at randomly selected system resource utilization levels. It is obvious from these two graphs that the transaction execution time increased when the scale of device groups expanded. However, the response graph was steady and the overall transaction execution capability could be assessed if no network congestion happened. Bitcoin takes 10 minutes to mine a blockchain; however, a bitcoin transaction generally needs six confirmations before it is finalized. As a result, it can be expected that a transaction takes around an hour on average, which is unbearable to the general public. Ethereum transaction times are around 15 seconds but the average time would increase exponentially according to varied network environments. The experiment results from the case study indicate that the proposed blockchain platform outperforms most other popular blockchain systems in terms of transaction time. The limitation of this work is that the experiment given was built on a limited size network, with only four peers. However, this is just a small case in order to prove the usability of the designed approach. As we mentioned earlier, the proposed architecture is adopted in a modular design that would be easy to extend. This can be achieved by adding a large number of peers in the network, as the Hyperledger Fabric provides the scripts to construct the underlying structure of the business network. 

**Figure 17:**
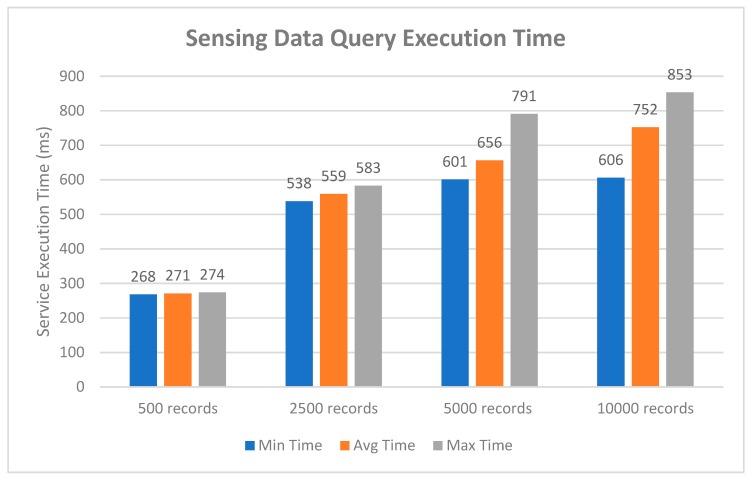
Performance analysis graph of the sensing data query.

For the third study, we evaluated the system performance when querying sensing records stored from the distributed ledger in the blockchain network. Figure 17 measured the execution time to query data records from the blockchain by varying the amount of data from 500 to 10,000 records. The minimum, average, and maximum delay time in ms taken by the proposed platform to retrieve the sensing records were noted down ten times at randomly selected system resource utilization levels. For the worst-case performance, with 10,000 records set, the minimum delay was recorded to be 606 ms, averaging at 752 ms, and the maximum delay was recorded to be 853 ms. It can be seen from the figure that the size of data records had great influence on the round-trip latency. However, the increase was maintained at such a low level that it could even be ignored, in other words, the impact on the user’s experience could be neglected.

## 6. Comparison and Significance

The following section conducts a comparative analysis of the proposed platform with some of the current studies that have been reviewed in the related work. In order to demonstrate the efficiency and capability of the designed platform, a benchmark study was carried out, and the evaluation results are presented in Table 8. 

**Figure 18:**
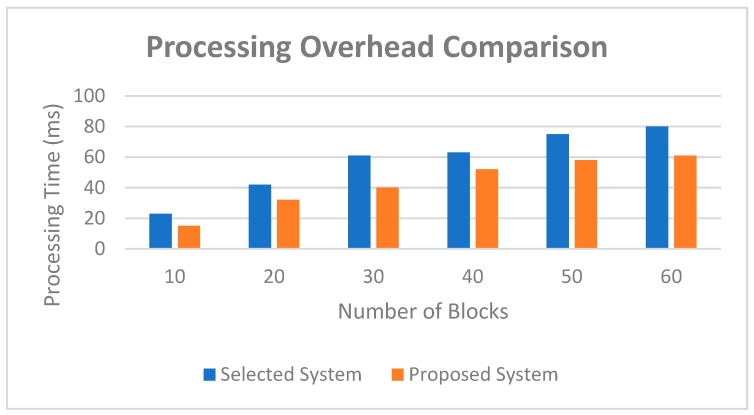
Performance analysis graph of processing overhead comparison.

The following properties that play a pivotal role to compare the overviewed platforms are considered for this study. Additionally, it reflects the overall performance of blockchain platform and highlights the significance of our proposed approach. As shown from the table, the presented system in [43] is a somewhat similar approach, with the most similar characteristics to our proposed work. As a result, we chose to compare this system with our proposed approach. The simulation environment for this analysis was set up the same as the selected system in [43]. We simulated a network of 50 peers and ran the simulation for 60 s, during which 960 transactions were executed. The processing time metric refers to the time cost on verifying new blocks in the network. Simulation results for evaluating the processing overhead are presented in Figure 18. As shown in the graph, the processing overhead with our approach was lower than the selected system by varying the number of blocks from 10 to 60. In general, our approach decreased the processing time by 22%.

It is obvious to see that most systems are built on a permissionless blockchain network, which allows anyone to participate, and every participant is anonymous. This means that neither can there be confidentiality of the contracts themselves, nor of the transaction data that they process. In order to mitigate the lack of confidentiality, these systems issue their own tokens to incent costly mining or to fuel smart contract execution. The transaction cost and transaction speed can be greatly affected by negative associations with cryptocurrencies. In addition, it obstructs the interaction with other distributed systems, as the token used in both systems must be unified. In contrast, the proposed system is built on a permissioned network, which diminishes the risk of a participant intentionally introducing malicious code through the smart contract. These participants are known to each other and all the actions are recorded on the blockchain in terms of the endorsement policy that was established for the network and transaction type. Furthermore, most existing systems lack the support of resource-constrained IoT devices, since they simply deploy full nodes on these devices to perform time-consuming mining. However, the resource-constrained architecture of IoT has always been the main hindrance in integrating IoT with the blockchain, since the consensus algorithms have to be limited to work within these constraints. Some current works deploy heavy consensus algorithms on other devices that are part of the IoT system, such as gateways. However, IoT gateways are generally small devices lack storage space. Many blockchain platforms do not yet provide support for lightweight nodes, and full nodes with an entire blockchain (more than 46 GB in Ethereum) must be deployed on the gateway for validation of transactions and blocks. Furthermore, this makes gateways, themselves, targets and also the first line of defense, since they act as bridges between devices and the Internet. The proposed solution, however, presents a lightweight solution that avoids integrating blockchain technology into IoT devices and these devices do not need any modification. The blockchain is used as an external service to provide a reliable and secure storage. Besides, the transactions made by IoT devices are validated in the blockchain network without downloading the entire blockchain. This improves the usability of our solution in a large variety of IoT scenarios with limited capabilities. In addition, the communication between IoT devices and the blockchain network happens through web service APIs, which enable cross platform communication. This approach also makes it possible to integrate with existing systems.

This paper presents a real-life case study for smart space, which was implemented as part of the experimental test in order to demonstrate the feasibility of the proposed system. This system is built on a modular architecture that can be easily extended to meet all kinds of requirements in various application domains, such as supply chain, energy trading, and data marketplace. For example, the proposed system can be expanded in the food supply chain to improve transparency and efficiency, since the blockchain technologies can provide a trusted source of information and traceability across the food network. IoT sensors can be attached to any product, like fish that is relegated to someone for transport, with remotely sensed data such as temperature, humidity, and location. By making a shared ledger accessible to each party in the supply chain, all food processing steps can be recorded and stored on the blockchain, including digital compliance documentation, test results, and audit certificates. The demand for an IoT blockchain application that offers a permissioned network, no currency exchange, friendly interface, flexible architecture, low latency of transaction, and high transaction throughput is high, and this work aims to look for ways to solve all these issues mentioned above.

## 7. Conclusions

With billions of connected devices coming online, there are systemic challenges to scaling IoT. Connected devices are always diverse and different from factors and manufactures. Therefore, identity and interoperability need to be assured in a secure manner. Furthermore, centralized architecture like the cloud model can have high costs, latency, and the risk of single point of failure. Blockchain technologies provide a new security protocol and infrastructure to enable billions of IoT devices to have trusted interoperability for both data and commerce. This paper outlines a novel approach for the design and implementation of a decentralized IoT platform to address scalability, identity, and data security challenges based on a permissioned blockchain network. A proof of concept of the proposed approach is implemented by using the Raspberry Pi and various physical devices. We evaluate the performance of the proposed system in various performance metrics, which indicated a steady level, allowing effective transaction execution. Furthermore, a comparative analysis of the designed system with existing works is performed to highlight the significance of this system in variety of aspects. Although the coevolution of blockchain and IoT research studies is still in its infancy, this work explores the potential applications of IoT and blockchain to improve efficiency and bring automation, to revolutionize robust business solutions in various IoT scenarios. Future research directions aim at testing the interoperability of the proposed system with different IoT frameworks. Furthermore, we are planning to test other consensus algorithms and data storage technologies in order to improve the transaction processing rate and make data query more efficient. 

## Figures and Tables

**Table 1:** Development environment for the IoT blockchain network.

Component	Description
CPU	Intel Core i5-8500 @ 3.00 GHz
Memory	12 GB
Operating Systems	Ubuntu Linux 18.04.1 LTS
Docker Engine	Version 18.06.1-ce
Docker-Compose	Version 1.13.0
Node	v8.11.4
Hyperledger Fabric	v1.2
IDE	composer-playground
CLI Tool	composer-cli, composer-rest-server
DBMS	Couch DB
Programming Language	Node.js

**Table 2:** Development environment for the Raspberry-based IoT device server.

Component	Description
Hardware	Raspberry Pi3 Model B
Memory	1 GB
Operating Systems	Android Things v0.8
Server	CoAP Server
Resources	Temperature, Humidity, Green LED, Red LED
IDE	Android Studio 3.1.4
Library and Framework	Californium CoAP, HttpURLConnection
Programming Language	Java

**Table 3:** Development environment for the blockchain web app.

Component	Description
Operating System	Windows 10 Pro 64 bit
Server	Apache Tomcat
IDE	Eclipse Photon (4.8.0), WebStorm (2018.2.3)
Browser	Chrome, Firefox, IE
Library and Framework	Californium CoAP, Notify.js, Bootstrap, jQuery
Programming Language	Java, HTML, CSS, JavaScript

**Table 4:** Device asset definition in the smart contract.

Category	Component	Type
Sensor	sensor_ID	String
	name	String
	device_owner	String
	unit	String
	event_threshold	Integer
	timestamp	DateTime
	value	String
Actuator	actuator_ID	String
	name	String
	device_owner	String
	state	Boolean

**Table 5:** Sample transaction definition in the smart contract.

Component	Type	Participant	Condition
Sensor reading	Transaction	Sensor	Asset = Sensor
Actuator writing	Transaction	Actuator	Asset = Actuator
Device creating	Transaction	Device owner	Participant = Device owner
Device updating	Transaction	Device owner	Participant ID = Device owner ID in device asset
Device deleting	Transaction	Device owner	Participant ID = Device owner ID in device asset

**Table 6:** Sample event definition in the smart contract.

Component	Type	Role
Sensor event	Event	Give notice when sensing value exceeds the threshold
Actuator event	Event	Give notice whenever the actuator state is changed
Device creating event	Event	Give notice when new device is added
Device updating event	Event	Give notice when specific device is updated
Device delete event	Event	Give notice when specific device is deleted

**Table 7:** RESTful API used for interaction.

URI	Verb	Media Type	Action
/api/DeviceOwner	ALL	Application/json	Device Owner Management
/api/Sensor	ALL	Application/json	Sensor Management
/api/Actuator	ALL	Application/json	Actuator Management
/api/Task	ALL	Application/json	Task Management
/api/SensorReading	GET, POST	Application/json	Add Sensing Data, Retrieve Sensing Data Log
/api/ActuatorWriting	GET, POST	Application/json	Update Actuator State, Retrieve Actuator State Log
/api/system/historian	GET	Application/json	Retrieve All Historian Records
/api/system/identities	GET	Application/json	Get All Identities
/api/systemidentities/issue	POST	Application/json	Issue an Identity to the Specific Participant
/api/system/ping	GET	Application/json	Test the Connection to the Blockchain Network

**Table 8:** Comparative study of the proposed IoT blockchain platform with existing platforms.

Name	Native Cryptocurrency	Consensus Determination	Mining Required	Smart Contract	Device as Node	Access Policy	Support Client
[36]	Yes	All Nodes	Yes	No	Yes	Permissionless	Yes
[37]	Yes	All Nodes	Yes	Yes	No	Permissionless	No
[41]	Yes	All Nodes	Yes	Yes	Yes	Permissionless	Yes
[43]	No	Arbitrary Nodes	No	Yes	No	Permissionless/Permissioned	Yes
[45]	Yes	All Nodes	Yes	Yes	Yes	Permissionless	Yes
[46]	Yes	All Nodes	Yes	Yes	Yes	Permissionless	Yes
[47]	Yes	All Nodes	Yes	No	No	Permissionless	Yes
[48]	Yes	All Nodes	Yes	No	Yes	Permissionless	Yes
[49]	No	All Nodes	Yes	Yes	Yes	Permissionless	Yes
[50]	Yes	All Nodes	Yes	Yes	No	Permissionless	Yes
[51]	Yes	All Nodes	Yes	Yes	No	Permissionless	Yes
Proposed Platform	No	Arbitrary Nodes	No	Yes	No	Permissioned	Yes
